# Gain of CXCR7 function with mesenchymal stem cell therapy ameliorates experimental arthritis via enhancing tissue regeneration and immunomodulation

**DOI:** 10.1186/s13287-021-02402-w

**Published:** 2021-05-29

**Authors:** Sung-Tai Wei, Yen-Chih Huang, Jung-Ying Chiang, Chia-Ching Lin, Yu-Jung Lin, Woei-Cherng Shyu, Hui-Chen Chen, Chia-Hung Hsieh

**Affiliations:** 1 Graduate Institute of Biomedical Sciences, China Medical University, Taichung, Taiwan; 2 Department of Neurosurgery, China Medical University and Hospital, Taichung, Taiwan; 3 Department of Medical Imaging, China Medical University and Hospital, Taichung, Taiwan; 4 Department of Medical Research, China Medical University Hospital, Taichung, Taiwan; 5 Department of Biomedical Informatics, Asia University, Taichung, Taiwan

**Keywords:** Mesenchymal stem cells, CXC chemokine receptor 7, rheumatoid arthritis, differentiation, immunomodulation

## Abstract

**Background:**

The major barriers to mesenchymal stem cell (MSC) therapy in rheumatoid arthritis (RA) are a low extent of tissue regeneration and insufficient immunomodulation after cell transplantation. In addition, the role of C-X-C chemokine receptor type 7 (CXCR7) and its mechanism of action in MSC-mediated osteogenic or chondrogenic differentiation and immunomodulation are unclear.

**Methods:**

Gain of CXCR7 function on human MSCs was carried out by lentiviral vector-mediated CXCR7 overexpression or CXCR7 agonist, TC14012. These cells were determined the role and potential mechanisms for CXCR7-regulated MSC differentiation and immunomodulation using cellular and molecular assays. The therapeutic benefits in RA were investigated in rats with collagen-induced arthritis (CIA).

**Results:**

CXCR7 was upregulated in MSCs during the induction of osteogenic or chondrogenic differentiation. Blockage of CXCR7 function inhibited osteogenic or chondrogenic differentiation of MSCs whereas gain of CXCR7 function had the opposite effects. Besides, MSCs with CXCR7 gain-of-function facilitated macrophage apoptosis and regulatory T cell differentiation in a co-culture system. Gain of CXCR7 function also promoted the production of anti-inflammatory soluble factors. A gene expression profiling assay and signaling reporter assays revealed that CXCR7 could regulate several candidate genes related to the PPAR, WNT, Hedgehog or Notch pathways, and their signaling activities, which are known to control cell differentiation and immunomodulation. Finally, MSCs with CXCR7 gain-of-function significantly reduced the articular index scores, ankle circumference, radiographic scores, histologic scores, and inflammation in rats with CIA compared with control MSCs.

**Conclusions:**

CXCR7 promotes the osteogenic and chondrogenic differentiation of MSCs and MSC-mediated immunomodulation by regulating several signaling pathways and anti-inflammatory soluble factors. MSCs with CXCR7 gain-of-function significantly ameliorate arthritic symptoms in a CIA model.

**Supplementary Information:**

The online version contains supplementary material available at 10.1186/s13287-021-02402-w.

## Introduction

Rheumatoid arthritis (RA) is a chronic symmetrical autoimmune disease caused by loss of immunologic self-tolerance and characterized by chronic joint inflammation and destruction [1]. Recently, significant advances in our understanding of the immune mechanisms in RA have driven the development of targeted biological therapies to block cytokines or pathogenic cells. However, these therapies are not curative for all RA patients, and their effectiveness is dependent on the improvement of inflammation or over-active immune response. This disadvantage has led to redirecting scientific efforts and resources towards the investigation of other therapeutic approaches. Stem cells have attracted much attention as potential therapeutic agents in the treatment of RA through replacing damaged cells, inducing endogenous tissue repair or modulating immunity [2]. However, clinical studies have shown that mesenchymal stem cell (MSC) treatment in RA patients resulted in only partial and transient clinical improvement [3]. Therefore, enhancement of stem cell function holds great promise for improving efficiency and clinical outcomes in RA.

The therapeutic efficacy of MSCs in tissue repair and regeneration is limited by several factors, including low survival, engraftment, and homing to the damage area as well as inefficiencies in differentiating into fully functional tissues [4, 5]. Although MSCs have immunosuppressive and anti-inflammatory properties, insufficient, and variable immunomodulatory potency of MSCs represents a failure to suppress inflammation or over-active immunity in autoimmune diseases [6–8]. To improve MSC therapy, the genetic engineering of MSCs, thereby combining MSCs with specific target agonists or antagonists, or preconditioning treatment serves as a powerful technology to enhance their therapeutic abilities [9]. Recent studies have shown that engineering of MSCs enhances their abilities in homing, survival, engraftment, differentiation, paracrine effects, and immunomodulation, resulting in positive effects in the case of several diseases. However, it is difficult to utilize single genetic manipulation in MSCs in order to address all the insufficient functions.

A growing body of evidence has demonstrated that an orphan receptor, RDC-1, now known as CXCR7, has multiple roles in cells [10]. Gain of CXCR7 function inhibits apoptosis and increases proliferation of cancer cells, endothelial cells, and stem cells whereas loss of CXCR7 function leads to the opposite [11]. CXCR7 plays a role in adhesion and migration depending on the particular cell type. It has been reported that CXCR4 is required for MSC migration and adhesion, whereas CXCR7 is responsible for MSC adhesion and survival [12]. Recently, CXCR7 has also been suggested to regulate differentiation of MSCs into type II alveolar epithelial cells and increase the ability of MSCs to enact anti-inflammation measures in rats with phosgene-induced acute lung injury [13]. These findings indicate that CXCR7 may be a potential target for MSCs to improve their ability to be therapeutic agents in autoimmune diseases.

In this study, we explored the role and mechanisms of CXCR7 regulation of osteogenic or chondrogenic differentiation and immunomodulation in human MSCs. With this, MSCs with CXCR7 gain-of-function were generated by genetic or pharmacological manipulation, and their therapeutic efficiency was evaluated in rats with collagen-induced arthritis (CIA).

## Materials and methods

### Cell cultures and MSC priming

Primary bone marrow-derived human MSCs were purchased from Sigma-Aldrich. MSCs were cultured in basic medium (α-MEM; Gibco) supplemented with 10% fetal bovine serum (FBS; Gibco), 100 U/ml penicillin (Gibco), 10 μg/ml streptomycin (Gibco), 2 mM l-glutamine (Gibco), and 0.2 mM l-ascorbic acid 2-phosphate magnesium salt (ASAp, Sigma-Aldrich) at 37°C in a humid atmosphere with 5% CO_2_. Human monocytic THP-1 cells were maintained in culture in Roswell Park Memorial Institute medium (RPMI 1640; Invitrogen) culture medium containing 10% heat-inactivated FBS (Invitrogen) and supplemented with 10 mM HEPES (Gibco), 1 mM pyruvate (Gibco), 2.5 g/l d-glucose (Merck), and 50 pM ß-mercaptoethanol (Gibco). THP-1 monocytes are differentiated into M1 macrophages by 24 h incubation with 150 nM phorbol 12-myristate 13-acetate (PMA; Sigma-Aldrich) followed by 24 h incubation in RPMI medium containing 20 ng/ml of IFN-γ (R&D Systems) and 10 pg/ml of lipopolysaccharides (LPS; Sigma-Aldrich). Jurkat-T cells were maintained in RPMI 1640 (Invitrogen) supplemented with 10% heat-inactivated FBS, 100 U/ml penicillin, and 100 mg/ml streptomycin (Gibco) at 37°C and 5% CO_2_. For MSC priming, MSCs were incubated with the media containing CCX771 (100 nM; ChemoCentryx) or TC14012 (30 μM; Sigma-Aldrich) for 48 h and collected for cell transplantation.

### MSCs characterization

To characterize MSCs, cells were incubated with 1 μg of phycoerythrin (PE)-conjugated antibodies (CD44, CD73, CD90, and CD105; BD Bioscience) or isotype-matched negative control antibody at 4°C for 45 min according to manufacturer’s instructions. Samples were analyzed by a flow cytometric analysis. Besides, MSCs were induced osteogensis, chondrogensis, and adiopgensis by using STEMPRO® osteogenesis, chondrogenesis, and adipogenesis differentiation kits (GIBCO). Medium was replaced every 4–7 days. Differentiation was terminated after 21 days. After the appearance of morphologic features of differentiation, cells were stained with Alizarin Red, Alcian Blue, and Oil Red for osteocytes, chondrocytes, and adiopocytes, respectively, using a Human Mesenchymal Stem Cell Differentiation kit (Thermo Fisher Scientific, Inc.), in accordance with the manufacturer’s protocol.

### Growth factors and inhibitors

Osteogenic and chondrogenic differentiation media were purchased from Lonza. TC14012 was obtained from Sigma-Aldrich. CCX771 was a kind gift from Dr. Mark Penfold (ChemoCentryx).

### Quantitative real-time polymerase chain reaction (Q-PCR)

Total RNA from the cells was obtained using an RNeasy Mini Kit (Qiagen) according to the manufacturer’s protocol and reverse-transcribed with Omniscript RT (Qiagen) using random hexamers (Applied Biosystems). Quantitative PCR was performed in an Opticon 2 Monitor (MJ Research) and SYBR Green I dye (Applied Biosystems) (for Q-PCR primer sequences, see Supplementary Table S1). The average of each gene cycle threshold (Ct) was determined for each experiment. Relative cDNA levels (2^−ΔΔCt^) for the genes of interest were determined using the comparative Ct method, which generates ΔΔCt as the difference between the gene of interest and the housekeeping gene 18S rRNA for each sample. Each averaged experimental gene expression sample was compared with the averaged control sample, which was set to 1.

### Flow cytometric analysis

Surface expression of CXCR7 or CXCR4 was evaluated by flow cytometric analysis. Cells were harvested with phosphate-buffered saline (PBS) containing 5 mM EDTA and immediately neutralized in fluorescence-activated cell sorting (FACS) buffer (α-MEM containing 1% BSA and 0.025% NaN_3_). After extensive washing with FACS buffer, cells (10^5^ cells) were incubated with 1 μg/ml of the primary antibodies, including CXCR7 and CXCR4 (R&D Systems), in a 1:100 dilution by shaking for 1 h at 4°C. After extensive washing with FACS buffer, cells were incubated with DyLight 649 AffiniPure goat anti-rabbit IgG or DyLight 488 AffiniPure goat anti-mouse IgG (Jackson Immunoresearch, 1:100 dilutions) by shaking for 1 h at 4°C. Cells were then washed with FACS buffer five to six times and fixed in PBS containing 1% paraformaldehyde. Besides, cell suspension derived from synovial tissue digestion was separated into different tubes and stained with FITC-CD11b or PE-Foxp3 to a determine the frequency of CD11b^+^ macrophages and Foxp3^+^ Treg cells in synovial tissues. Expression levels were measured on a FACScalibur instrument and with FACSDiva 6.0 software (BD Bioscience).

### Plasmids

To construct pAS2.CXCR7-Puro-H, total human RNA was extracted from MCF7 cells using the RNeasy kit (Qiagen). A total of 500 ng RNA was used in a reverse transcription reaction, and cDNA was generated with Superscript II reverse transcriptase (Invitrogen). Full-length human CXCR7 cDNA was amplified in a reaction with Platinum Taq DNA polymerase (Invitrogen) using the human CXCR7 primers as described previously [14, 15], which harbored 5′ NheI and 3′ and EcoRI sites. The fragments were subcloned into pAS2.EYFP.puro (National RNAi core facility, Academia Sinica, Taiwan) at the NheI and EcoRI sites, respectively, and then the cDNA sequences were confirmed. Lentiviral vectors carrying short hairpin RNAs (shRNAs)-targeting CXCR4 and scrambled shRNA (http://rnai.genmed.sinica.edu.tw/file/vector/C6-7/17.1.pLAS.Void.pdf) were provided by the National RNAi core facility, Academia Sinica, in Taiwan. The detailed shRNA target sequences used in this study are described in Supplementary Table S2.

### Lentivirus production and transduction

Lentiviral particles were generated by transiently co-transfecting 293T cells with the plasmids coding for CXCR7 (pAS2.CXCR7-Puro-H), scrambled shRNA, and shRNAs-targeting CXCR4 in addition to plasmids encoding gag/pol and VSV-G envelope genes. Transfection was carried out with jetPEI reagent (Polyplus-Transfection). Subconfluent cells were infected with lentivirus in the presence of 8 μg/ml polybrene (Sigma-Aldrich). At 24 h post-infection, the medium was removed and replaced with fresh growth medium containing puromycin (0.5 ug/ml) select for infected cells after 48 h post-infection.

### Western blot analysis

Cell extracts were prepared as described previously [16]. Besides, the fractionation of cytoplasm or nucleus protein was isolated by using the subcellular protein fractionation kit (Thermo Scientific) according to the manufacturer’s recommended procedures. A total of 30 ug of protein was loaded and electrophoresed using SDS-PAGE gel and then transferred to a polyvinylidene difluoride (PVDF) membrane. The membrane was blocked for 1 h using blocking solution and was then incubated with the primary antibody overnight at 4°C. The following antibodies were used: β-actin (Sigma-Aldrich, 1:10000 dilution), osteopontin (R&D Systems, 1:500 dilution), aggrecan (Sigma-Aldrich, 1:200 dilution), PPAR-γ (Thermo Fisher Scientific, 1:200 dilution), β-catenin (abcam, 1:100), glioma-associated oncogene homolog 1 (Gli1) (Cell Signaling Technology, 1:200 dilution) and intracellular domain of the notch protein (NICD) (Cell Signaling Technology, 1:200 dilution). On the second day, after three washing steps with TBS-0.05% Tween-20, the blot was incubated with secondary horseradish peroxidase-conjugated antibody (Sigma-Aldrich, 1:10000 dilution) for 45 min. The blot was next washed three times with TBS-0.05% Tween-20; then, a super signal west pico chemiluminescent substrate (Thermo Scientific) was applied for the detection of protein bands. Relative band densities of the various proteins were measured from scanned films using ImageJ Software (NIH).

### Apoptosis assay

Cocultures of human M1 macrophages or Jurkat T cells and MSCs were harvested after 1 and 3 days. Apoptosis of macrophages or regulatory T cells was quantified by FACS after incubation with a monoclonal human CD11b-PE antibody (Thermo Fisher Scientific-eBioscience) to discriminate between macrophages and MSCs or a monoclonal human FoxP3-PE antibody (Thermo Fisher Scientific-eBioscience) to discriminate between regulatory T cells and MSCs, followed by the annexin V-FITC method (Abcam).

### Enzyme-linked immunosorbent assay (ELISA)

ELISAs were performed to evaluate interleukin (IL)-6, IL-10, leukemia inhibitory factor (LIF), prostaglandin E2 (PEG2), nitric oxide (NO), tumor necrosis factor (TNF)-α, IL-1β, and receptor activator of nuclear factor kappa-Β ligand (RANKL), osteocalcin, and type I collagen cross-linked C-telopeptide (CTX-I) levels in the conditional medium (CM) or tissue homogenates according to the manufacturer’s instructions (Thermo Fisher Scientific-eBioscience and Novus Biologicals).

### Gene expression profiling assay

A Human Signal Transduction PathwayFinder™ RT2 Profiler™ PCR Array was used to screen a panel of 84 genes representative of 10 different signal transduction pathways in MSCs with or without CXCR7 overexpression according to the manufacturer’s instruction (Qiagen).

### Signaling reporter assay

MSCs were co-transfected with peroxisome proliferator responsive element (PPRE-luciferase reporter vector, TCF/LEF luciferase reporter vector, CSL (CBF1/RBP-Jκ) luciferase reporter vector, 8X Gli luciferase reporter vector, or pRL-TK Renilla luciferase control plasmids (Promega) using Lipofectamine stem transfection reagent (Thermo Fisher Scientific-eBioscience). Luciferase activity was assayed at 48 h after transfection with the Dual-Glo luciferase assay system (Promega). Values corresponding to the ratio of firefly to renilla luciferase activity were used to calculate signaling activity.

### Rat collagen-induced arthritis (CIA)

The animal model of CIA was established according to the previously described protocol [17]. Male Sprague–Dawley rats (8 weeks old) were immunized with an emulsion composed of equal parts of Freund’s complete adjuvant (FCA) containing 4 mg/ml heat-killed Mycobacterium tuberculosis (Chondrex) and bovine type II collagen (CII) solubilized at 2 mg/ml in 0.05 M acetic acid. The rats received an intradermal injection of 200 μl of emulsion (200 μg of bovine CII) into the dorsum. Booster doses were administered on day 8, with subcutaneous 100-μl injections of the same emulsion into the base of the tail. The onset of CIA was between days 12 and 16 after the first immunization.

### MSC treatments

All animal studies were conducted according to the Institutional Guidelines of China Medical University and approved by the Institutional Animal Care and Use Committees of China Medical University (Approval Number: 102-92-N). Treatment was begun after the onset of disease (day 16). Rats received a single intraarticular injection at a dose of 2 × 10^6^ cells native MSCs, control vector expressing MSCs (control MSCs), CXCR7-expressing MSCs (CXCR7-MSCs), vehicle-primed MSCs, CCX771-primed MSCs, or TC14012-primed MSCs in the hind paw with arthritic symptoms. PBS was used as a control for MSC injection. All rats were sacrificed 7 weeks after the first immunization, and joint tissues were collected for further studies.

### Clinical and radiographic assessments

Hind paw swelling and ankle width were measured every day and performed as blind experiments. Articular index was dependent on hind paw swelling severity, with grading from 0 to 4. The center of foot maximal swelling was measured as paw thickness by dial caliper. The formula of ankle circumference was 2*π*√(a^2^ **+** b^2^/2), a: laterolateral diameter; and b: anteroposterior diameter. Radiographs of the hand paw were taken with an X-ray instrument (40 kV, 100 mA, 6/100 s; Toshiba, MRAD-A50S).

### Histology

The ankle joints were dissected from sacrificed animals at the end of the study and fixed in 4% formaldehyde at 4°C overnight and then decalcified in ethylenediaminetetraacetic acid buffer for 4 weeks following the protocol for paraffin embedding. The microtomy was performed with slices of 7 μm. The slides were stained with hematoxylin and eosin (H&E) for general morphological tissue analysis, and with Safranin-O fast green staining for analysis of articular cartilage. All sections were evaluated histologically by two independent observers. The histological score of arthritis ranged from 0 to 4 according to the intensity of lining layer hyperplasia, mononuclear cell infiltration, and pannus formation, as described previously [18]: 0, normal ankle joint; 1, normal synovium with occasional mononuclear cells; 2, definite arthritis, a few layers of flat to rounded synovial lining cells and scattered mononuclear cells; 3, clear hyperplasia of the synovium with three or more layers of loosely arranged lining cells and dense infiltration with mononuclear cells; and 4, severe synovitis with pannus and erosions of articular cartilage and subchondral bone. Safranin-O staining was scored with a semiquantitative scoring system (0–3), where 0 represents no loss of proteoglycans and 3 indicates complete loss of staining for proteoglycans [19].

### Synovial tissue digestion

Synovial tissues were digested with 4 ml collagenase type II, which was dissolved in serum free DMEM, for 1h at 37 °C. Then, cell suspension was centrifuged at 300 g (1290 rpm) for 10 min at 4 °C and the pellet was incubated with 0.25% pancreatin for 30 min at 37°C. The pellet was resuspended with PBS. Cell suspension was filtered by 200 μm nylon mesh and stained before a flow cytometer analysis.

### Statistical analysis

All data are supplied as mean ± standard deviation (SD). Statistical analyses were performed with the Statistical Package for the Social Sciences (SPSS), version 18.0 (IBM, Chicago, USA) software using unpaired Student’s t test and analysis of variance (ANOVA) with Bonferroni’s or Tukey’s multiple comparison post hoc tests where appropriate.

## Results

### CXCR7 is upregulated in MSCs during the induction of osteogenic and chondrogenic differentiation

**Fig. 1:**
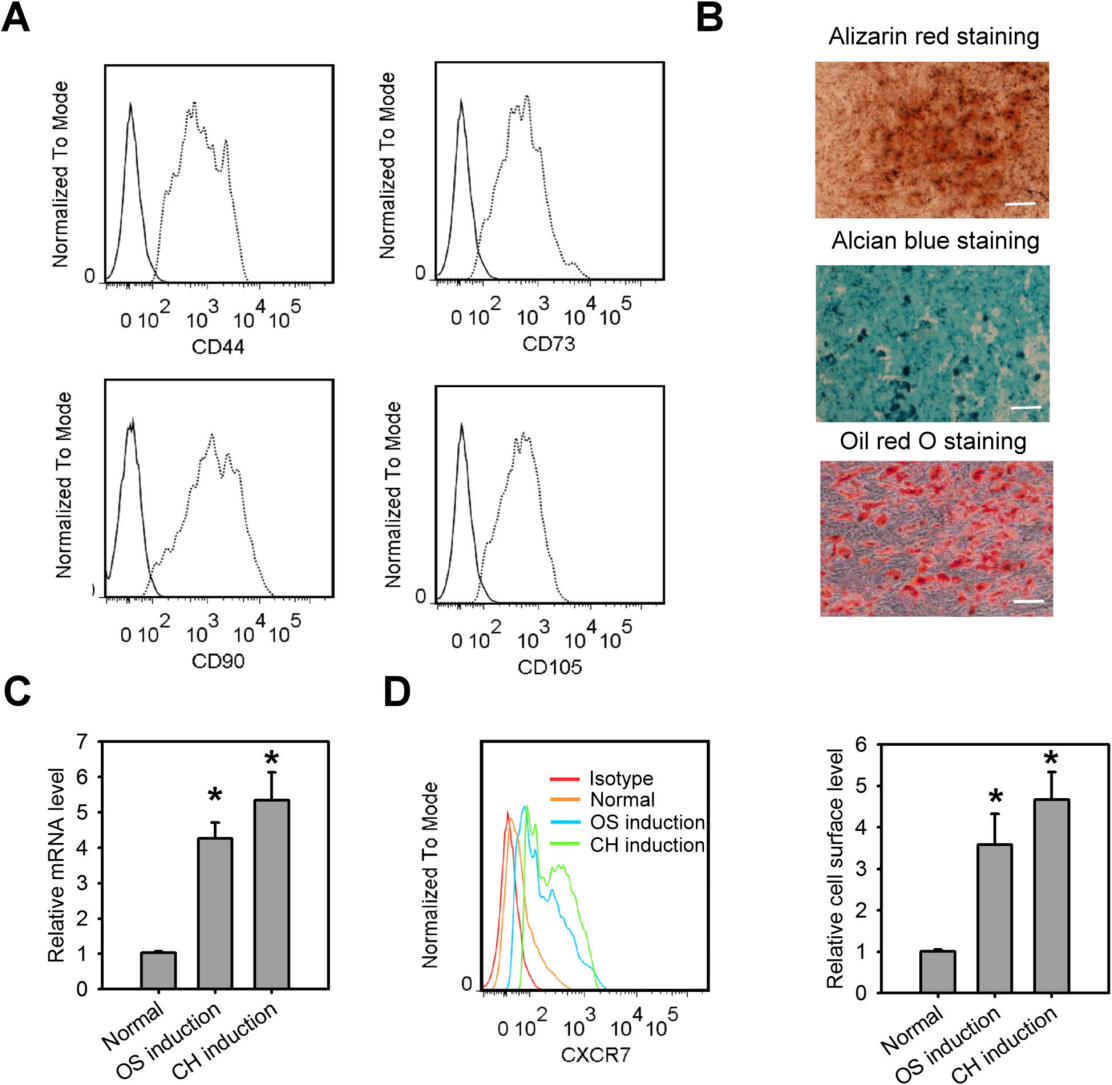
CXCR7 is upregulated in MSCs during the induction of osteogenic and chondrogenic differentiation. **a** Cell surface co-expression of the antigens, CD44, CD73, CD90, and CD105 in MSCs. **b** Differentiation potential of MSCs in osteogenic, chrondrogenic, and adipogenic lineages using Alizarin red, Alcian blue, and oil red O staining, respectively. The mRNA levels (**c**) and cell-surface expression (**d**) CXCR7 in MSCs incubated with osteogenic or chondrogenic induction medium for 48 h. Data are means ± SD (*n*=9). *P < 0.0001 compared to the normal (untreated) group

The MSCs used in our study highly expressed CD73, CD44, CD105, and CD90 (Fig. 1a) and had the potential to differentiate along osteogenic, chondrogenic, and adipogenic lineages (Fig. 1b). We first observed the expression characteristics of CXCR7 in the osteogenic and chondrogenic differentiation of MSCs. Q-PCR and flow cytometry revealed that CXCR7 was less expressed in MSCs with normal culture medium (Fig. 1c, d). However, the transcript and surface levels of CXCR7 were increased greatly in osteogenic or chondrogenic differentiation medium-treated MSCs, with the peak of expression at days 2–3 after the culture on the induction mediums, which lasted for up to 21 days (Supplemental Fig. 1a), indicating the induction of osteogenic or chondrogenic differentiation is able to trigger CXCR7 expression on MSCs.

### CXCR7 contributes to osteogenic and chondrogenic differentiation of MSCs

**Fig. 2:**
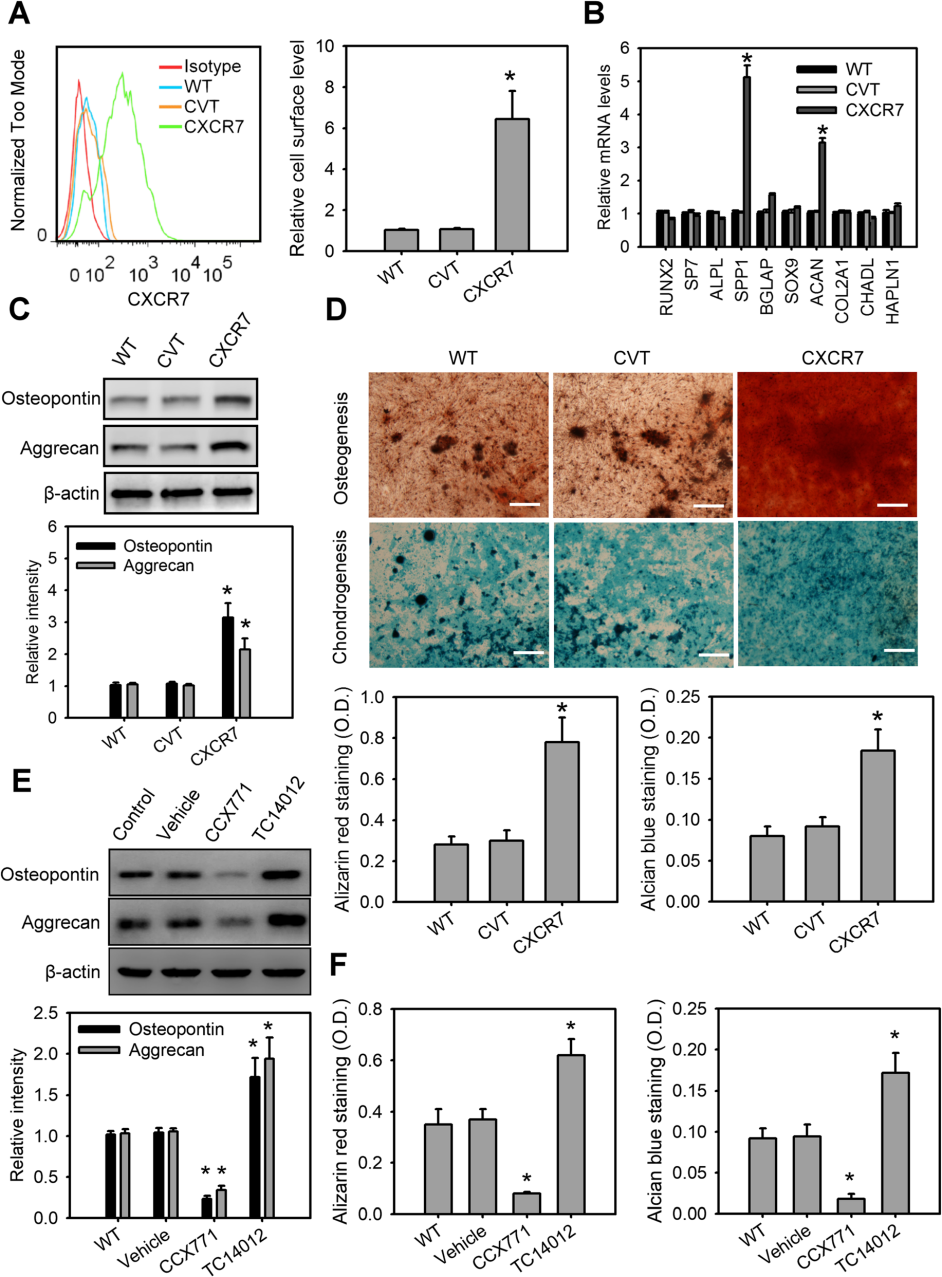
CXCR7 contributes to osteogenic and chondrogenic differentiation of MSCs. **a** Verification of CXCR7 overexpression in MSCs lentivirally transduced with control vector (CVT) or CXCR7-expressing vector (CXCR7) for 72 h. Data are means ± SD (*n*=9). *P < 0.0001 compared with wild-type MSCs (WT). **b** Changes in osteogenic and chondrogenic genes in MSCs with or without CXCR7 overexpression incubated with osteogenic or chondrogenic induction medium for 72 h. Data are means ± SD (*n*=9). *P < 0.0001 compared with wild-type MSCs (WT). **c** The protein levels of osteopontin and aggrecan in MSCs with or without CXCR7 overexpression incubated with osteogenic or chondrogenic induction medium for 72 h. Data are means ± SD (*n*=9). *P < 0.001 compared with wild-type MSCs (WT). **d** Alizarin red and Alcian blue staining and their quantitation in MSCs with or without CXCR7 overexpression incubated with osteogenic or chondrogenic induction medium for 21 days. Data are means ± SD (*n*=9). *P < 0.001 compared with wild-type MSCs (WT). The protein levels of osteopontin or aggrecan (**e**) and quantitation of Alizarin red and Alcian blue staining (**f**) in MSCs with or without the CXCR7 antagonist, CCX771, or agonist, TC14012, incubated with osteogenic or chondrogenic induction medium for 72 h and 21 days, respectively. Data are means ± SD (*n*=9). *P < 0.001 compared with the untreated group (WT)

We next tested the role of CXCR7 in osteogenic and chondrogenic differentiation of MSCs. MSCs were stably transduced using recombinant lentiviruses expressing CXCR7. Flow cytometric analysis confirmed successful CXCR7 overexpression in MSCs (Fig. 2a). CXCR7 overexpression on MSCs could slightly decrease the expression of CXCR4 (Supplemental Fig. 2a). Q-PCR was utilized to determine the changes of osteogenic genes, such as runt-related transcription factor 2 (RUNX2), osteoblast-specific transcription factor (SP7), alkaline phosphatase (ALPL), osteopontin (SPP1) and osteocalcin (BGLAP), and chondrogenic genes, such as SOX-9 (SOX9), aggrecan (ACAN), type II collagen (COL2A1), chondroadherin-like protein (CHADL), and hyaluronan and proteoglycan link protein 1 (HAPLN1) in MSCs with or without CXCR7 overexpression during the treatment with osteogenic or chondrogenic differentiation medium (Fig. 2b). CXCR7 overexpression in MSCs promoted the expression of SPP1 (a marker of osteoblastic cell differentiation) and ACAN (a marker of chondrogenic cell differentiation) compared to control lentiviral vector-infected MSCs or wild-type MSCs. Western blot analysis also confirmed the protein levels of osteopontin and aggrecan highly expressed in MSCs with CXCR7 overexpression (Fig. 2c). Moreover, MSCs with CXCR7 overexpression showed the strong positive staining for Alizarin red and Alcian blue dyes and elevation of ALPL, SPP1, ACAN, and COL2A1 expression at 21 days of osteogenic or chondrogenic induction compared to control lentiviral vector-infected MSCs or wild-type MSCs (Fig. 2d and Supplemental Fig. 1b). Interestingly, treatment of MSCs with a CXCR7 antagonist, CCX771, inhibited osteogenic or chondrogenic differentiation of MSCs whereas treatment of MSCs with CXCR7 agonist, TC14012, had the opposite effect (Fig. 2e, f and Supplemental Fig. 1c). In order to rule out the potential effect derived from CXCR7 gain-of-function-mediated downregulation of CXCR4 on MSCs, we knocked down CXCR4 in MSCs using CXCR4 target shRNA via a lentiviral-based system. However, no significance difference was observed in MSCs with or without CXCR4 knockdown (Supplemental Fig. 2a-e), suggesting CXCR7, but not CXCR4, promotes osteogenic or chondrogenic differentiation of MSCs.

### CXCR7 enhanced immunosuppression of MSCs

**Fig. 3:**
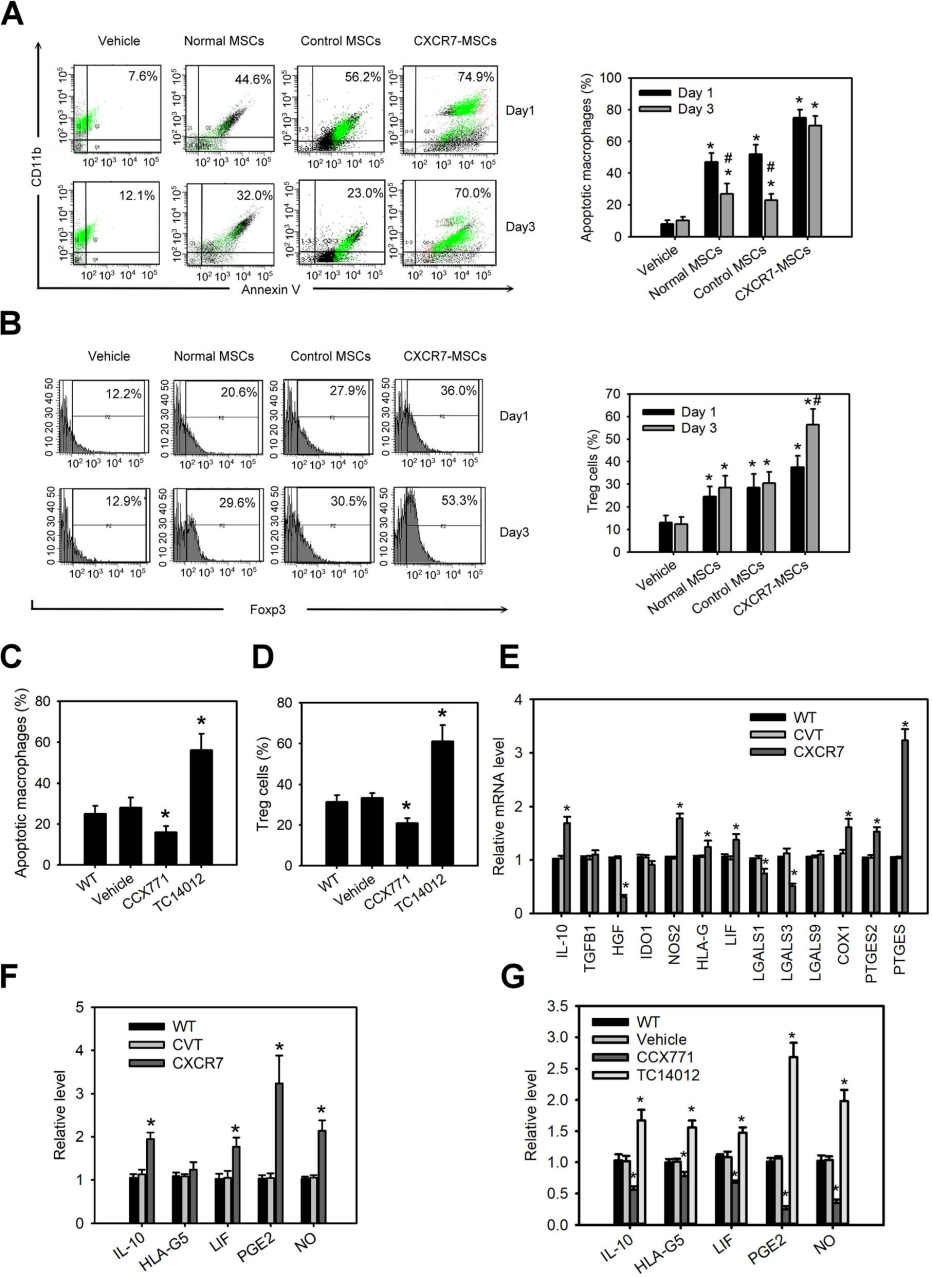
CXCR7 enhanced immunosuppression of MSCs. **a** Macrophage apoptosis of LPS-induced THP-1 macrophages (CD11b^+^ cells) co-cultured with MSCs with or without CXCR7 overexpression for 1 or 3 days. Data are means ± SD (*n*=9). *P < 0.0001 compared with the vehicle-treated group. ^#^ *P* < 0.001 compared with the groups on day 1. **b** Differentiation of regulatory T (Treg, FoxP3^+^ cells)-like cells in Jurkat T cells co-cultured with MSCs with or without CXCR7 overexpression for 1 or 3 days. Data are means ± SD (*n*=9). *P < 0.001 compared with the vehicle-treated group. ^#^ *P* < 0.001 compared with the groups on day 1. **c** Macrophage apoptosis of LPS-induced THP-1 macrophages co-cultured with MSCs with or without the CXCR7 antagonist, CCX771, or agonist, TC14012, treatment for 3 days. Data are means ± SD (*n*=9). *P < 0.001 compared with the untreated group (WT). **d** Differentiation of regulatory T-like cells in Jurkat T cells co-cultured with MSCs with or without CCX771 or TC14012 treatment for 3 days. Data are means ± SD (*n*=9). *P < 0.001 compared the untreated group (WT). **e** Changes in the expression of genes associated with soluble factors secreted by MSCs with or without CXCR7 overexpression during immunomodulation. Data are means ± SD (*n*=6). *P < 0.001 compared the control MSCs without lentiviral transduction (WT). **f** The protein levels of IL-10, LIF, PGE2, and NO in the condition medium of MSCs with or without CXCR7 overexpression. Data are means ± SD (*n*=6). *P < 0.0001 compared with the control MSCs without lentiviral transduction (WT). **g** Protein levels of IL-10, LIF, PGE2, and NO in the condition medium of MSCs with or without CCX771 or TC14012 treatment for 3 days. Data are means ± SD (*n*=6). *P < 0.001 compared with the untreated group (WT)

Macrophages and regulatory T (Treg) cells are critically involved in the pathogenesis of RA [20, 21]. MSCs are able to induce the immunosuppression via regulating these immune cells [22]. Therefore, we next focused on investigating the cell-to-cell interactions between MSCs and these immune cells. MSCs were co-cultured with LPS-induced THP-1 macrophages or Jurkat T cells for 1 and 3 days. LPS-induced THP-1 macrophages co-cultured with wild-type MSCs exhibited an increase in macrophage apoptosis (Fig. 3a). Moreover, macrophage apoptosis was greatly elevated in the co-culture with CXCR7-overexpressing MSCs compared with that in the co-culture with control MSCs or wild-type MSCs. Interestingly, a significant decrease in macrophage apoptosis was observed at day 3 after co-culturing with control MSCs or wild-type MSCs whereas co-culturing with CXCR7-overexpressing MSCs did not uncover such an effect. In contrast to macrophages, Jurkat T cells co-cultured with wild-type MSCs showed an increase in regulatory T (Treg, FoxP3^+^ cells)-like cells (Fig. 3b). Jurkat T cells co-cultured with CXCR7-overexpressing MSCs experienced more expansion in Treg-like cells than in Jurkat T cells co-cultured with control MSCs or wild-type MSCs. Moreover, treatment of MSCs with TC14012 also had a similar effect whereas MSCs with CCX771 treatment generated the opposite effects (Fig. 3c, d). MSCs with CXCR4 knockdown co-cultured with LPS-induced THP-1 macrophages or Jurkat T cells did not have the significant effects in macrophage apoptosis or regulatory T expansion (Supplemental Fig. 2f and g). Further, we also used Q-PCR to screen the genes associated with soluble factors secreted by MSCs in immunomodulation. Among these genes, IL-10, nitric oxide synthase 2 (NOS2), HLA-G, LIF, cyclooxygenase 1 (COX1), prostaglandin E synthase 2 (PTGES2), and prostaglandin E synthase (PTGES) were significantly upregulated in MSCs with CXCR7 overexpression whereas hepatocyte growth factor (HGF), galectin-1 (LGALS1), and galectin-3 (LGALS3) were downregulated (Fig. 3e). The ELISA also confirmed that the levels of IL-10, LIF, PGE2, and NO in condition medium of CXCR7-overexpressing MSCs or TC14012-treated MSCs were significantly higher than those in the medium of control MSCs, vehicle-treated MSCs, or wild-type MSCs but the opposite appeared in CCX771-treated MSCs (Fig. 3f, g). These findings indicate CXCR7 plays a role in MSC-mediated immunosuppression by regulating anti-inflammatory soluble factors.

### CXCR7 upregulates PPAR, WNT, Hedgehog, and Notch signaling on MSCs

**Fig. 4:**
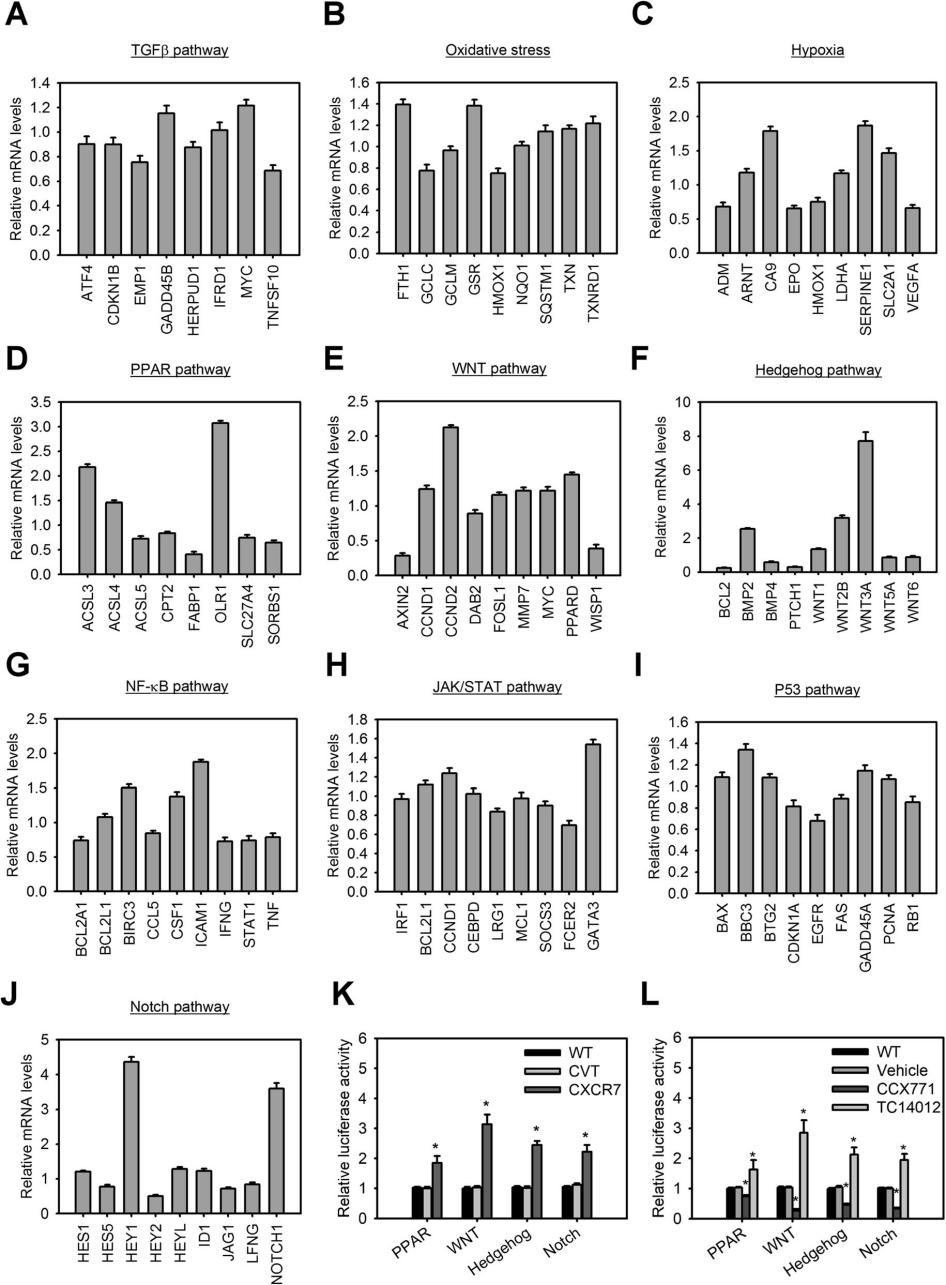
CXCR7 upregulates PPAR, WNT, Hedgehog, and Notch signaling on MSCs**.** Up- and downregulated mRNA profiling in terms of TGF-β (**a**), oxidative stress (**b**), hypoxia (**c**), PPAR (**d**), WNT (**e**), Hedgehog (**f**), NF-κB (**g**), JAK/STAT (**h**), P53 (**i**), and Notch (**j**) signaling pathways for MSCs with or without CXCR7 overexpression using the Human Signal Transduction Pathway Finder RT2 Profiler™ PCR Array. **k** PPAR, WNT, Hedgehog and Notch signaling activities in MSCs with or without CXCR7 overexpression using the reporter assays. Data are means ± SD (*n*=6). *P < 0.001 compared with native MSCs (WT). **l** PPAR, WNT, Hedgehog, and Notch signaling activities in MSCs with or without the CXCR7 antagonist, CCX771, or agonist, TC14012, treatment for 3 days. Data are means ± SD (*n*=6). *P < 0.001 compared with the untreated group (WT)

In order to explore the CXCR7-mediated potential pathways involved in the aforementioned phenotypes or mechanisms, we carried out gene expression profiling using the Human Signal Transduction Pathway Finder RT2 Profiler™ PCR Array. The expression changes of 84 key genes representing 10 signal transduction pathways in MSCs with or without CXCR7 overexpression were assessed. Among these pathways, there were more alterations in genes in the PPAR, WNT, Hedgehog, and Notch signaling pathways (Fig. 4a–j). The present study found that 14 out of the 84 examined genes changed in terms of at least a two-fold differential expression in CXCR7-overexpressed MSCs versus control MSCs. Moreover, the signaling reporter assays were further used to confirm their signaling activities. MSCs with CXCR7 overexpression significantly increased PPAR, WNT, Hedgehog, and Notch signaling activities compared to control MSCs or wild-type MSCs (Fig. 4k). MSCs with CXCR7 overexpression also increased the nuclear localization of their transcription factors such as PPAR-γ, β-catenin, Gli1, or NICD compared with control MSCs (Supplemental Fig. 3). Besides, treatment of MSCs with TC14012 also exhibited similar effects whereas MSCs with CCX771 treatment generated the opposite effects (Fig. 4l). These results suggest that CXCR7 is able to regulate PPAR, WNT, Hedgehog, and Notch signaling pathways.

### CXCR7 gain-of-function improves the therapeutic efficiency of MSCs in experimental arthritis

**Fig. 5:**
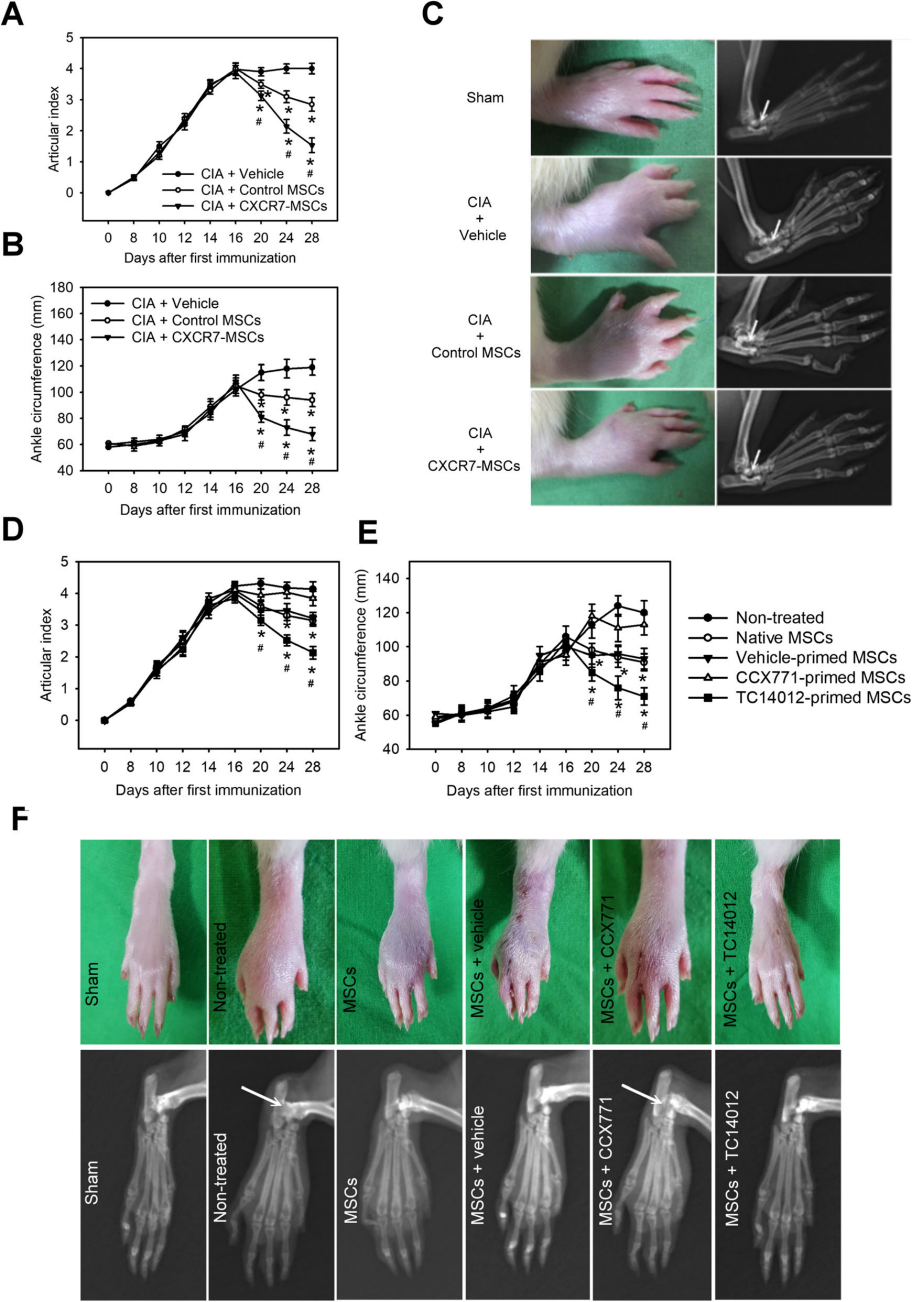
CXCR7 gain-of-function improves the therapeutic efficiency of MSCs in experimental arthritis. Articular index (**a**) and ankle circumference (**b**) in rat collagen-induced arthritis (CIA) with or without CXCR7-genetically engineered MSC treatments. Rats were immunized with collagen on days 0 and 7, followed by injection of control vector-expressing MSCs (control MSCs), CXCR7-expressing MSCs (CXCR7-MSCs), or vehicle (PBS) into each ankle joint on day 16. Data are means ± SD (*n*=6–8). *P < 0.01 compared with the vehicle treatment. ^#^ *P* < 0.01 compared with the control MSC treatment. **c** Representative morphologies and radiographs of the rat paw or ankles receiving the aforementioned treatments on day 28. Arrows indicate obvious differences in the degree of joint damage between groups. Articular index (**d**) and ankle circumference (**e**) in normal rat (sham) or rat with collagen-induced arthritis (CIA) receiving native MSC (MSCs), vehicle-primed MSC, CCX771-primed MSC, or TC14012-primed MSC treatment. Rats were immunized with collagen on days 0 and 7, followed by injection of CCX771, TC14012, or vehicle-primed MSCs into each ankle joint on day 16. Data are means ± SD (*n*=6–8). *P < 0.01 compared with the untreated group (non-treated). ^#^ *P* < 0.01 compared with the native MSC (MSCs) treatment. **f** Representative morphologies and radiographs of the rat paw or ankles receiving the aforementioned treatments on day 28. Arrows indicate obvious differences in the degree of joint damage between groups

We next observed the therapeutic efficiency of MSCs with CXCR7 gain-of-function in the rat CIA model. Rats were immunized with collagen on days 0 and 7, followed by injection of CXCR7-overexpressed MSCs, control MSCs, or vehicle (PBS) into each ankle joint on day 16. Compared with vehicle or control MSCs, CXCR7-overexpressed MSCs were significantly decreased with respect to articular index score and ankle circumference (Fig. 5a, b). Arthritic rats treated with CXCR7-overexpressed MSCs or control MSCs experienced significant inhibition in swelling of the ankles (Fig. 5c). Radiographic analysis of the ankle joint at 28 days after FCA injection also showed a marked suppression of joint destruction in the CXCR7-overexpressed MSCs or control MSCs, whereas a remarkable extent of bone destruction was observed among animals receiving vehicle treatment (Fig. 5c). Moreover, arthritic rats with CXCR7-overexpressed MSC treatment had less ankle swelling and radiographic features of joint destruction compared with the ankle joint injected with control MSCs, suggesting CXCR7-overexpressed MSCs are superior to control MSCs transduced with control vector for cell therapy of experimental arthritis. Of note, transplantation of TC14012-primed MSCs also had similar effects. However, the opposite was found in CCX771-primed MSC transplantation (Fig. 5d–f). These results suggest that CXCR7 gain-of-function is able to enhance the therapeutic efficiency of MSCs in experimental arthritis.

### MSCs with CXCR7 gain-of-function decrease the histopathological features and severity of arthritis

**Fig. 6:**
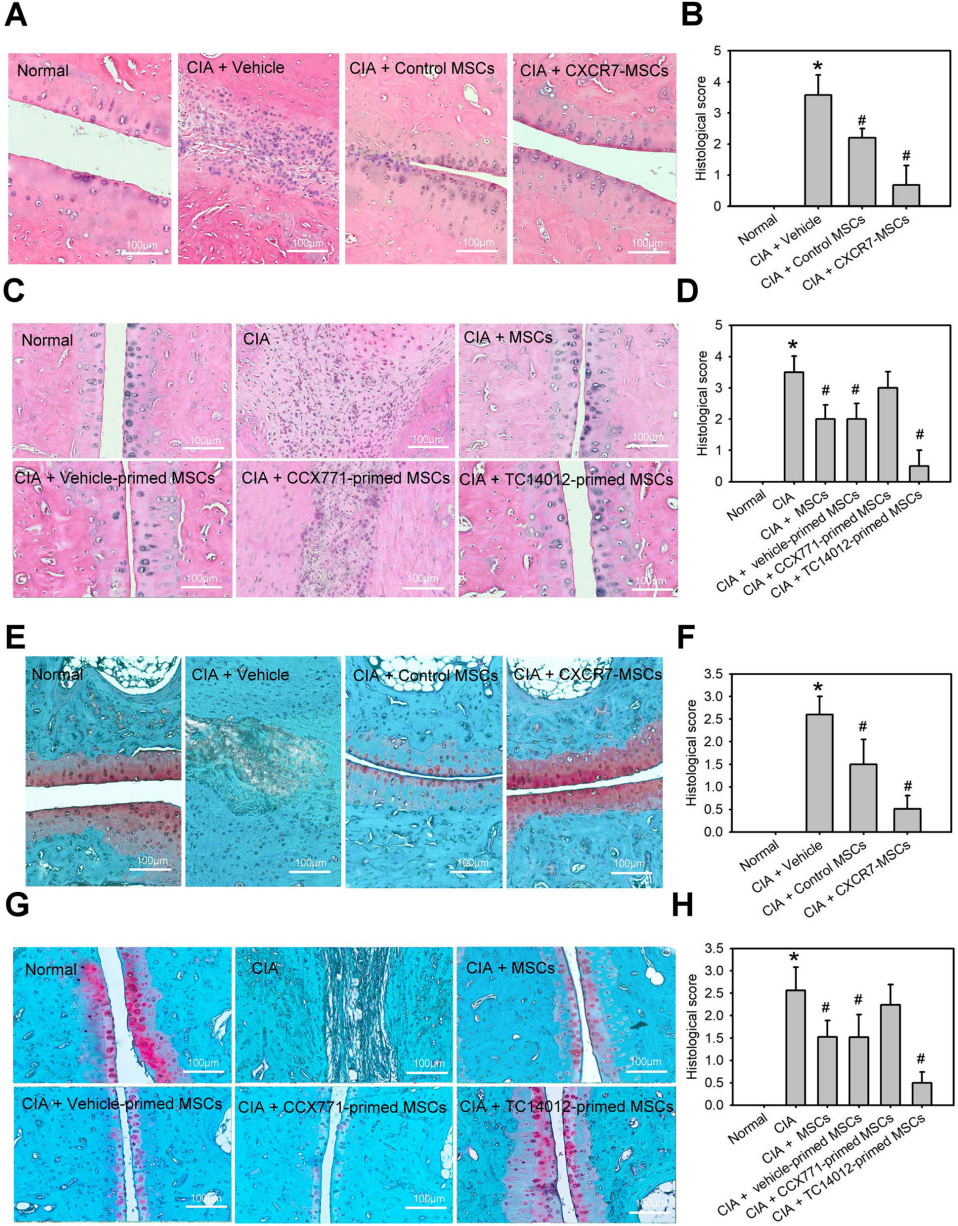
MSCs with CXCR7 gain-of-function decrease the histopathological features and severity of arthritis. **a**–**d** Representative histopathologies of the ankle joints stained with haematoxylin and eosin (H&E) and histologic joint scores in normal rats or rats with collagen-induced arthritis (CIA) receiving vehicle (PBS), control vector-expressing MSC (control MSCs), CXCR7-expressing MSC (CXCR7-MSCs), native MSCs (MSC), vehicle-primed MSC, CCX771-primed MSC, or TC14012-primed MSC treatment on day 28. Data are means ± SD (*n*=6–8). *P < 0.01 compared with the normal group without CIA. ^#^ *P* < 0.01 compared with the CIA group without any treatment. **e**-**h** Representative histopathologies of the ankle joints stained with Safranin-O and histologic joint scores in normal rats or rats with CIA receiving vehicle (PBS), control vector-expressing MSC (control MSCs), CXCR7-expressing MSC (CXCR7-MSCs), native MSC (MSCs), vehicle-primed MSC, CCX771-primed MSC, or TC14012-primed MSC treatment on day 28. Data are means ± SD (*n*=6–8). *P < 0.01 compared with the normal group without CIA. ^#^ *P* < 0.01 compared with the CIA group without any treatment

In order to confirm the improvement of clinical symptoms, histological evaluation of the joint tissues was performed. H&E staining analysis of vehicle-treated joint tissues revealed a marked infiltration of inflammatory cells, deformity of the ankle joints, an increase in the thickness of the synovial membrane, and severe bone destruction. These symptoms were significantly suppressed by administration of CXCR7-overexpressing MSCs, TC14012-primed hMSCs, control MSCs, or native MSCs (Fig. 6a–d). In the Safranin-O staining assay, minor positive staining with Safranin-O was observed in sections of synovial tissue examined in the vehicle-treated group, but the rounded morphology of mature chondrocytes was surrounded by deposited glycosaminoglycans (GAGs) (deep red color) in CXCR7-overexpressed MSCs, TC14012-primed MSCs, control MSCs, or native MSC-treated groups (Fig. 6e–h), indicating transplantation of MSCs inhibited cartilage damage. Moreover, the histopathological features and severity in CXCR7-overexpressed MSCs and the TC14012-primed MSCs group were significantly less than in control MSCs or native MSCs. However, transplantation of CCX771-primed MSCs also demonstrated opposite effects. Besides, the frequency of CD11b^+^ macrophages and Foxp3^+^ Treg cells in synovial tissues derived from normal rats or rats with CIA receiving vehicle, control vector-expressing MSC, or CXCR7-expressing MSC treatment on day 3 was analyzed by flow cytometry analysis. The high number of CD11b^+^ macrophages and low number of Foxp3^+^ Treg cells were found in synovial tissue suspensions of CIA rats (Supplemental Fig. 4). MSCs treatment substantially reduced the infiltration of macrophages and increased the amount of Treg cells into synovial tissues. CXCR7 overexpression on MSCs could enhance these effects. Taken together, these results suggest that MSCs with CXCR7 gain-of-function could enhance the improvement of histopathological features and severity of arthritis in a CIA model.

### MSCs with CXCR7 gain-of-function inhibit the molecular features and severity of arthritis

**Fig. 7:**
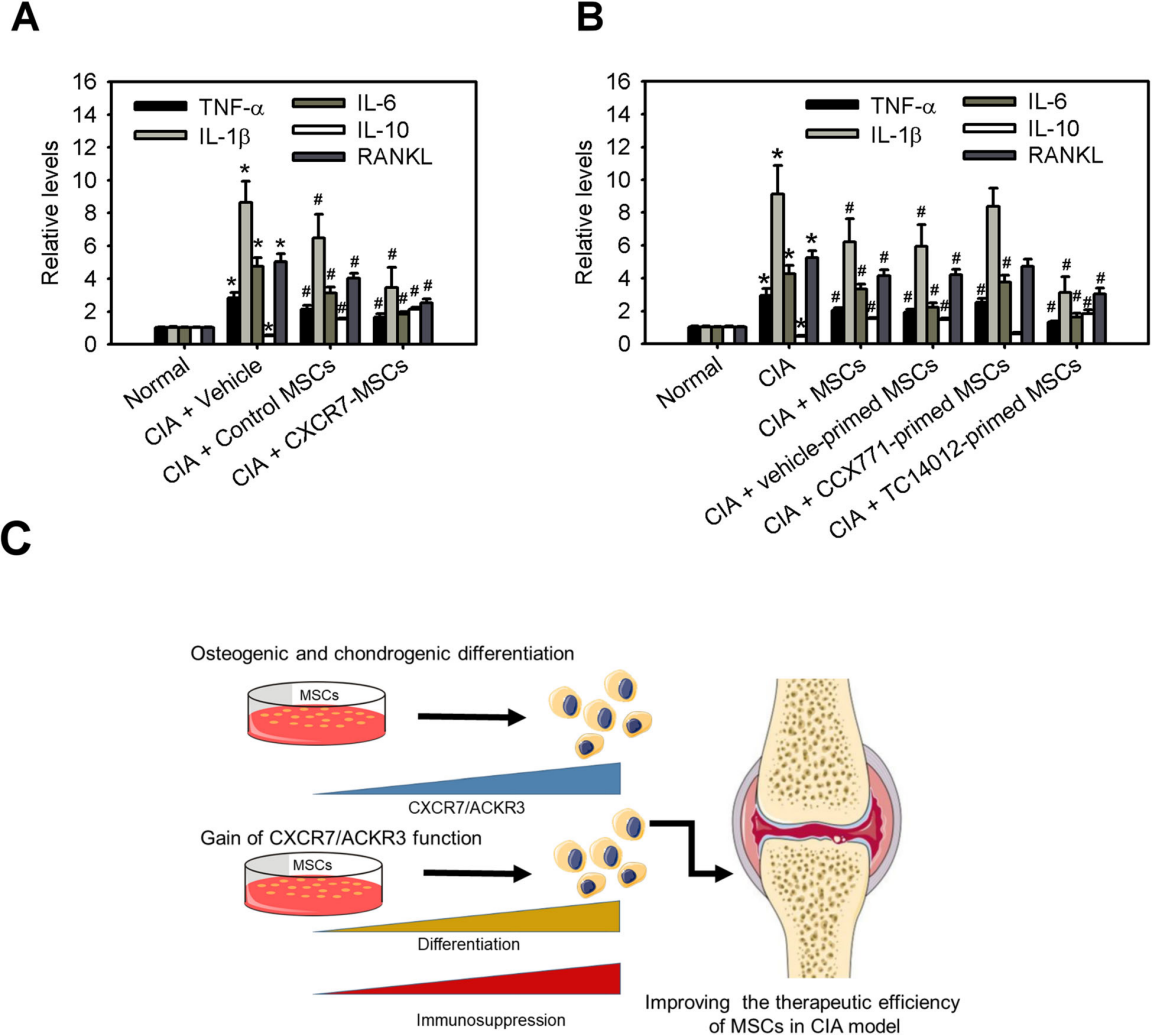
MSCs with CXCR7 gain-of-function inhibit the molecular features and severity of arthritis. **a**, **b** Levels of TNF-α, IL-1β, IL-6, IL-10, and RANKL in synovial tissues derived from normal rats or rats with collagen-induced arthritis (CIA) receiving vehicle (PBS), control vector-expressing MSC (control MSCs), CXCR7-expressing MSC (CXCR7-MSCs), native MSC (MSCs), vehicle-primed MSC, CCX771-primed MSC, or TC14012-primed MSC treatment on day 28. Data are means ± SD (*n*=6–8). *P < 0.01 compared with the normal group without CIA. ^#^ *P* < 0.01 compared with the CIA group without any treatment. **c** Working model of CXCR7 gain-of-function with mesenchymal stem cell therapy ameliorates experimental arthritis

Po-inflammatory cytokines, such as TNF-α, IL-1β, and IL-6, are considered pivotal cytokines in the pathogenesis of RA [23, 24]. In contrast, IL-10 is an anti-inflammatory cytokine that is produced by T helper type 2 (Th2) cells, activated monocytes/macrophages and certain Treg cells [25]. Further, RANKL is considered an important regulator of the promotion of excessive osteoclast activity and bone resorption or erosion in RA [26, 27]. Osteocaicin and CTX-I are the reference markers of bone formation and resorption, respectively, for monitoring therapy in clinical settings [28]. To observe the changes in these molecular features in arthritic rats with or without MSC treatments, we determined the expression of TNF-α, IL-1β, IL-6, IL-10, RANKL, osteocaicin, and CTX-I in knee synovial tissues from arthritic rats using ELISA. In the vehicle-treated group, CIA significantly increased the levels of TNF-α, IL-1β, IL-6, RANKL, and CTX-I but decreased IL-10 and osteocaicin levels in arthritic rats (Fig. 7a, b and Supplemental Fig. 5a-d). However, these effects were significantly inhibited by administration of MSCs. Moreover, the downregulation of TNF-α, IL-1β, IL-6, RANKL, and CTX-I as well as upregulation of IL-10 and osteocaicin in CXCR7-overexpressed MSCs or TC14012-primed MSCs were significantly greater than those in control MSCs or native MSCs, indicating that MSCs with CXCR7 gain-of-function effectively inhibited the production of pro-inflammatory cytokines and bone resorption and promoted bone formation. However, transplantation of CCX771-primed MSCs decreased these effects. Taken together, the results indicated overall that MSCs with CXCR7 gain-of-function are able to inhibit the molecular features and severity of arthritis by regulating the production of inflammatory cytokines and bone turnover in synovial tissues.

## Discussion

MSCs have been shown to inhibit arthritis in experimental animal models with CIA [29]. Moreover, the results from human clinical trials demonstrate RA patients with resistance to disease-modifying anti-rheumatic drugs (DMARDs) receiving allogeneic MSCs were safe but resulted in only partial clinical improvement [30]. Therefore, the strategy for enhancement of the therapeutic potential of MSCs is necessary to develop improved efficiency and clinical outcomes in RA. The results presented here provide compelling evidence that osteogenic or chondrogenic differentiation of MSCs can induce CXCR7 expression, and CXCR7 functions as a regulator of osteogenic or chondrogenic differentiation along with immunomodulation of MSCs in vitro. Moreover, MSCs with CXCR7 gain-of-function significantly decreased arthritic symptoms in a CIA model. These findings support a rationale that MSCs genetically engineered with CXCR7 or CXCR7 agonist-primed MSCs are able to enhance the therapeutic potential of MSCs in the treatment of RA (Fig. 7c).

The precise activity of CXCR7 in stem cell biology remains poorly understood. Previous studies have shown that overexpression of CXCR7 significantly increased the proliferation and migration of murine MSCs without any inflammatory response [31]. The potential mechanisms underlying this phenomenon may derive from CXCR7-mediated positive feedback regulation of the C–X–C motif chemokine ligand 12 (CXCL12) and is related to increased expression of vascular cell adhesion molecule-1 (VCAM-1), CD44, and matrix metalloproteinase-2 (MMP-2). Further, the CXCL12/CXCR4/CXCR7 axis also promotes human neural progenitor cell survival [32]. Both the CXCR4- and CXCR7-mediated extracellular signal-regulated kinases (ERK)1/2 endocytotic signaling pathways contribute to CXCL12-mediated anti-apoptosis. A recent study reported that overexpression of CXCR7 promoted the differentiation of murine MSCs into type II alveolar epithelial cells [13], suggesting CXCR7 plays a part in the differentiation of murine MSCs. In agreement with this finding, we found that CXCR7 is an essential regulator for osteogenic and chondrogenic differentiation of MSCs. Pharmacological blockade of CXCR7 inhibits osteogenic and chondrogenic differentiation of MSCs whereas CXCR7 gain-of-function has the opposite effects. Additional studies are necessary to verify in vivo role of CXCR7 in osteogenic and chondrogenic differentiation of MSCs and clarify whether CXCR7 can also regulate other cell specifications by MSCs.

Several studies have revealed that CXCR7 functions in the modulation of immune responses through controlling the differentiation and maturation of immune cells or the production and secretion of soluble factors in targeting cells. CXCR7 is induced during monocyte-to-macrophage differentiation, which contributes to macrophage phagocytosis [33]. Moreover, B cell differentiation and maturation also upregulate CXCR7 expression and further influence the migration of B cells via CXCR7 as a scavenger for CXCR7 [34]. This similar mechanism also limits B cells in the splenic marginal zone [35]. Besides, inflammation upregulates CXCR7 expression in endothelial cells, epithelial cells, tumor cells, or other target cells, which contributes to specific homing or niches of immune cells in several diseases [36–38]. Although CXCR7 functions as a scavenger for CXCL12 and further regulates CXCR4-mediated chemotaxis, CXCR7 also promotes the expression and secretion of several cytokines, such as C–C motif chemokine ligand 2 (CCL2) or IL-8, which induces recruitment and activation of immune cells [36, 39]. For CXCR7 as it pertains to MSC-mediated immunomodulation, it has been known that the CXCL12/CXCR7 axis with murine MSCs can induce IL-10-producing regulatory B cells and IL-10 secretion, which contributes to the generation of an immunosuppressive environment [40]. Here, we identified other soluble factors involved in MSC-mediated immunomodulation, among them being IL-10, LIF, PGE2, HLA-G5, and NO [41]. These soluble factors are known to contribute human MSC-induced macrophage plasticity or apoptosis and Treg generation [42, 43]. Our results also highlight that gain of CXCR7 function on MSCs is able to enhance immunosuppression in a CIA model, suggesting CXCR7 plays a role in human MSC-mediated immunomodulation.

CXCR7 can influence CXCL12/CXCR4-mediated signaling via either sequestering extracellular CXCL12 or modulating CXCR4 signaling by CXCR7/CXCR4 heterodimerization [10]. Moreover, CXCR7 is capable of activating the ERK, Akt, or MAPK pathways through β-arrestin [44, 45]. Therefore, it can be imagined that CXCR7-regluated signaling transduction is complex. Our gene expression profiling and signaling reporting data reflect this concept. We screened the expression changes of 84 key genes representing 10 signal transduction pathways in MSCs with or without CXCR7 overexpression and identified four CXCR7-regulated signaling pathways, including the PPAR, WNT, Hedgehog, and Notch signaling pathways. Although it is still unclear what the mechanisms underlying CXCR7 regulation of these signaling pathways are, these forms of signal transduction are known to contribute to the differentiation or immunomodulation of MSCs. With this, activation of PPAR, WNT, Hedgehog, or Notch signaling promotes osteogenic or chondrogenic differentiation of MSCs [46–49]. Besides, Notch signaling also promotes MSC expansion of regulatory dendritic or T cells [50, 51]. The Wnt/β-catenin pathways also induce IL-10 expression and secretion, which contributes to immune suppression via direct β-catenin/TCF binding to the IL-10 promoter [52]. These signaling pathways not only partially explain the potential mechanisms of CXCR7-regulated differentiation and immunomodulation of MSCs, but also provide a valid basis for exploration of the role and mechanism of CXCR7 in stem cell biology in the future.

Previous studies have suggested that the pathogenic role of CXCR7 in rheumatoid arthritis [53]. The elevation of CXCR7 expression was found in endothelial cells in human RA synovium. Moreover, mice with CIA received the treatment with CXCR7 inhibitor, CCX733, significantly reduced the arthritis scores and the numbers of vessels in the synovial tissues, suggesting CXCR7 is potential target for novel RA antiangiogenic therapy. However, these findings cannot affect our conclusions or clinical application because the target cells for CXCR7 manipulation are different. Besides, our findings provide a potential risk for the combination of CCX733 and MSCs in treatment of RA because inhibition of CXCR7 in MSCs can reduce their osteogenic or chondrogenic differentiation and immunosuppressive properties.

## Conclusion

These data provide conclusive evidence that osteogenic or chondrogenic differentiation of MSCs through induced CXCR7 expression plays an important role in promotion of the differentiation and immunomodulation of MSCs. Moreover, CXCR7 promotes anti-inflammatory soluble factors, such as IL-10, LIF, PGE2, HLA-G5, or NO and regulates the PPAR, WNT, Hedgehog, and Notch signaling pathways, which are known to be involved in the differentiation and immunomodulation of MSCs. Moreover, this work provides a proof-of-concept to support that transplantations of MSCs with either genetic modification or pharmacological precondition-induced CXCR7 gain-of-function, potentially enhancing therapeutic efficacy during the treatment of RA.

## Supplementary Information

**Additional file 1: Supplementary table S1.** Q-PCR primer sequences. **Supplementary table S2.** shRNA target sequences. **Supplemental figure 1 a** Time course of cell-surface expression of CXCR7 after exposure of MSCs to osteogenic (OS) or chondrogenic ( CH) differentiation medium. The cell-surface expression of CXCR7 was determined by flow cytometry. **b** Changes in osteogenic (ALPL, SPP1) and chondrogenic (ACAN, COL2A1) genes in MSCs with or without CXCR7 overexpression incubated with osteogenic or chondrogenic induction medium for 21 days. Data are means ± SD (*n*=9). *P < 0.0001 compared with wild-type MSCs (WT). **c** Changes in osteogenic (ALPL, SPP1) and chondrogenic (ACAN, COL2A1) genes in MSCs with or without the CXCR7 antagonist, CCX771, or agonist, TC14012, incubated with osteogenic or chondrogenic induction medium for 21 days, respectively. Data are means ± SD (*n*=9). *P < 0.0001 compared with the untreated group (control). **Supplemental figure 2**. **a** Cell-surface expression of CXCR7 and CXCR4 in MSCs lentivirally transduced with control vector (CVT) or CXCR7-expressing vector (CXCR7) for 72 h. Data are means ± SD (*n*=9). *P < 0.001 compared with CVT- expressing MSCs. **b** Verification of CXCR4 knockdown in MSCs lentivirally transduced with the CXCR4 shRNA for 72 h. Data are means ± SD (*n*=9). *P < 0.0001 compared with wild-type MSCs (control). **c** Changes in osteogenic (ALPL, SPP1) and chondrogenic (ACAN, COL2A1) genes in MSCs with or without CXCR4 knockdown incubated with osteogenic or chondrogenic induction medium for 21 days. Data are means ± SD (*n*=9). (**d** and **e**) The quantitation of Alizarin red and Alcian blue staining in MSCs with or without CXCR4 knockdown incubated with osteogenic or chondrogenic induction medium for 21 days. Data are means ± SD co-cultured with MSCs with or without CXCR4 knockdown for 1 or 3 days. Data are means ± SD (*n*=9). *P < 0.001 compared with the groups on Day 1. (G) Differentiation of regulatory T (Treg, FoxP3+ cells)-like cells in Jurkat T cells co-cultured with MSCs with or without CXCR4 knockdown for 1 or 3 days. Data are means ± SD (*n*=9). **Supplemental figure 3** The localization of PPAR-γ, β-catenin, Gli1 or NICD evaluated by cell fractionation. PPAR-γ, β-catenin, Gli1 and NICD in MSCs lentivirally transduced with control vector (CVT) or CXCR7-expressing vector (CXCR7) for 72 h were detected in both the cytoplasmic and nuclear fractions via western blot analysis. β-actin and Histone-3 were used as internal controls for protein expression in the cytoplasmic and nuclear fractions, respectively. Data are means ± SD (*n*=9). *P < 0.01 compared with control MSCs (CVT). **Supplemental figure 4.** The frenquency of CD11b+ macrophages (**a**) and Foxp3+ Treg cells (**b**) in synovial tissues derived from normal rats or rats with collagen-induced arthritis (CIA) receiving vehicle (PBS), control vector-expressing MSC (control MSCs) or CXCR7-expressing MSC (CXCR7-MSCs) treatment on day 3. The single cell suspension of synovial tissues were made by enzymatic isolation method and the frenquency of CD11b+ macrophages and Foxp3+ Treg cells were detected by flow cytometry. Data are means ± SD (*n*=6). *P < 0.01 compared with the normal group without CIA. # *P* < 0.01 compared with the control MSCs. **Supplemental figure 5.** Levels of osteocaicin (**a** and **b**) and type I collagen cross-linked C-telopeptide (CTX-I, **c** and **d**) in synovial tissues derived from normal rats or rats with collagen-induced arthritis (CIA) receiving vehicle (PBS), control vector-expressing MSC (control MSCs), CXCR7-expressing MSC (CXCR7-MSCs), native MSC (MSCs), vehicle-primed MSC, CCX771-primed MSC or TC14012-primed MSC treatment on Day 28. Data are means ± SD (*n*=6). *P < 0.01 compared with the normal group without CIA. # *P* < 0.01 compared with the CIA group without any treatment.

## Data Availability

The data that support the findings of this study are available from the corresponding author upon reasonable request.

## Abbreviations

MSC: Mesenchymal stem cell
RA: Rheumatoid arthritis
CXCR7: A C–X–C chemokine receptor type 7
CIA: Collagen-induced arthritis
FBS: Fetal bovine serum
ASAp: l-ascorbic acid 2-phosphate magnesium salt
RPMI: Roswell Park Memorial Institute medium
PMA: Phorbol 12-myristate 13-acetate
LPS: Lipopolysaccharides
Q-PCR: Quantitative real-time polymerase chain reaction
IL: Interleukin
LIF: Leukemia inhibitory factor
PEG2: Prostaglandin E2
NO: Nitric oxide
TNF: Tumor necrosis factor
RANKL: Receptor activator of nuclear factor kappa-Β ligand
CM: Conditional medium
RUNX2: Runt-related transcription factor 2
SP7: Osteoblast-specific transcription factor
ALPL: Alkaline phosphatase
SPP1: Osteopontin
BGLAP: Osteocalcin
SOX9: SOX-9
ACAN: Aggrecan
COL2A1: Type II collagen
CHADL: Chondroadherin-like protein
HAPLN1: Hyaluronan and proteoglycan link protein 1
Treg: Regulatory T cell
NOS2: Nitric oxide synthase 2
COX1: Cyclooxygenase 1
PTGES2: Prostaglandin E synthase 2
PTGES: Prostaglandin E synthase
HGF: Hepatocyte growth factor
LGALS1: Galectin-1
LGALS3: Galectin-3
H&E: Hematoxylin and eosin
Th2: T helper type 2
DMARDs: Disease-modifying anti-rheumatic drugs
CXCL12: C–X–C motif chemokine ligand 12
VCAM-1: Vascular cell adhesion molecule-1
MMP-2: Matrix metalloproteinase-2
ERK: Extracellular signal-regulated kinases
CCL2: C–C motif chemokine ligand 2
CTX-I: Type I collagen cross-linked C-telopeptide
Treg: Regulatory T cells
GLI1: Glioma-associated oncogene homolog 1
NICD: Intracellular domain of the notch protein
