# Supplementary material for: Gain of CXCR7 function with mesenchymal stem cell therapy ameliorates experimental arthritis via enhancing tissue regeneration and immunomodulation
Source: Stem Cell Res Ther. 2021 May 29;12:314. doi: 10.1186/s13287-021-02402-w (PMC8164772; doi:10.1186/s13287-021-02402-w)
Supplement: Supplementary file 1 — Additional file 1: Supplementary table S1. Q-PCR primer sequences. Supplementary table S2. shRNA target sequences. Supplemental figure 1 a Time course of cell-surface expression of CXCR7 after exposure of MSCs to osteogenic (OS) or chondrogenic ( CH) differentiation medium. The cell-surface expression of CXCR7 was determined by flow cytometry. b Changes in osteogenic (ALPL, SPP1) and chondrogenic (ACAN, COL2A1) genes in MSCs with or without CXCR7 overexpression incubated with osteogenic or chondrogenic induction medium for 21 days. Data are means ± SD (n=9). *P < 0.0001 compared with wild-type MSCs (WT). c Changes in osteogenic (ALPL, SPP1) and chondrogenic (ACAN, COL2A1) genes in MSCs with or without the CXCR7 antagonist, CCX771, or agonist, TC14012, incubated with osteogenic or chondrogenic induction medium for 21 days, respectively. Data are means ± SD (n=9). *P < 0.0001 compared with the untreated group (control). Supplemental figure 2. a Cell-surface expression of CXCR7 and CXCR4 in MSCs lentivirally transduced with control vector (CVT) or CXCR7-expressing vector (CXCR7) for 72 h. Data are means ± SD (n=9). *P < 0.001 compared with CVT- expressing MSCs. b Verification of CXCR4 knockdown in MSCs lentivirally transduced with the CXCR4 shRNA for 72 h. Data are means ± SD (n=9). *P < 0.0001 compared with wild-type MSCs (control). c Changes in osteogenic (ALPL, SPP1) and chondrogenic (ACAN, COL2A1) genes in MSCs with or without CXCR4 knockdown incubated with osteogenic or chondrogenic induction medium for 21 days. Data are means ± SD (n=9). (d and e) The quantitation of Alizarin red and Alcian blue staining in MSCs with or without CXCR4 knockdown incubated with osteogenic or chondrogenic induction medium for 21 days. Data are means ± SD co-cultured with MSCs with or without CXCR4 knockdown for 1 or 3 days. Data are means ± SD (n=9). *P < 0.001 compared with the groups on Day 1. (G) Differentiation of regulatory T (Treg, FoxP3+ cells)-like cells in Jurkat T cells co- [file 13287_2021_2402_MOESM1_ESM.pdf]

SUPPLEMENTAL MATERIAL

Gain of CXCR7 function with mesenchymal stem cell therapy ameliorates experimental arthritis via enhancing tissue regeneration and immunomodulation

Sung-Tai Wei[1,2,+], Yen-Chih Huang[1,3,+], Jung-Ying Chiang[1,2], Chia-Ching Lin[1], Yu-Jung Lin[1], Woei-Cherng Shyu[1], Hui-Chen Chen[1], Chia-Hung Hsieh[1,4,5,*]

[1]Graduate Institute of Biomedical Sciences, China Medical University, Taichung, Taiwan

[2]Department of Neurosurgery, China Medical University and Hospital, Taichung, Taiwan

[3]Department of Medical Imaging, China Medical University and Hospital, Taichung, Taiwan

[4]Department of Medical Research, China Medical University Hospital, Taichung, Taiwan

[5]Department of Biomedical Informatics, Asia University, Taichung, Taiwan

[+] these authors contributed equally to this work

Supplementary table S1.

Primer	Sequence	Size(bp)
RUNX2	F: TCAACGATCTGAGATTTGTGGG<br>R: GGGGAGGATTTGTGAAGACGG	81
SP7	F: CCTCTGCGGGACTCAACAAC<br>R: AGCCCATTAGTGCTTGTAAAGG	128
ALPL	F: ACCACCACGAGAGTGAACCA<br>R: CGTTGTCTGAGTACCAGTCCC	79
SPP1	F: GGAGTTGAATGGTGCATACAAGG<br>R: CCACGGCTGTCCCAATCAG	75
BGLAP	F: GGCGCTACCTGTATCAATGG<br>R: GTGGTCAGCCAACTCGTCA	110
SOX9	F: AGCGAACGCACATCAAGAC<br>R: CTGTAGGCGATCTGTTGGGG	85
ACAN	F: ACTCTGGGTTTTCGTGACTCT<br>R: ACACTCAGCGAGTTGTCATGG	81
COL2A1	F: TGGACGCCATGAAGGTTTTCT<br>R: TGGGAGCCAGATTGTCATCTC	183
CHADL	F: GCCTGATCTACCTGTACCTCTC<br>R: CAGGAAACGGTTGCGTTCTA	110
HAPLN1	F: TATCGAGACCCTACAGCATTTGG<br>R: AGGTAATCCGAAGTTAGCTTGGT	77
IL-6	F: AGACAGCCACTCACCTCTTCAG<br>R: TTCTGCCAGTGCCTCTTTGCTG	132
IL-10	F: TCTCCGAGATGCCTTCAGCAGA<br>R: TCAGACAAGGCTTGGCAACCCA	126
TGFB1	F: TACCTGAACCCGTGTTGCTCTC<br>R: GTTGCTGAGGTATCGCCAGGAA	122
HGF	F: CGTAGCGTACCTCTGGATTGC<br>R: CGTAGCGTACCTCTGGATTGC	99
IDO1	F: TGGGGCAAAGGTCATGGAG<br>R: TTTCTTGGAGAGTTGGCAGTAAG	78

Supplementary table S2. shRNA target sequences

Gene	Target sequence
Human CXCR4-1	AGATAACTACACCGAGGAAAT
Human CXCR4-2	TCCTGTCCTGCTATTGCATTA

Supplemental figure 1

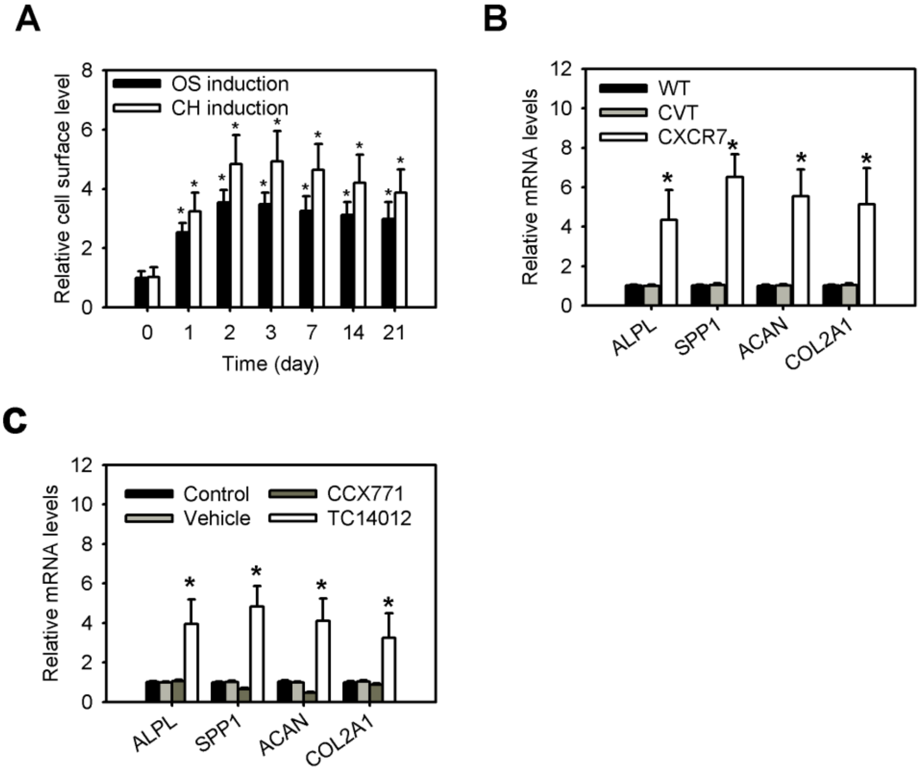

Supplemental figure 1. **a** Time course of cell-surface expression of CXCR7 after exposure of MSCs to osteogenic (OS) or chondrogenic (CH) differentiation medium. The cell-surface expression of CXCR7 was determined by flow cytometry. **b** Changes in osteogenic (ALPL, SPP1) and chondrogenic (ACAN, COL2A1) genes in MSCs with or without CXCR7 overexpression incubated with osteogenic or chondrogenic induction medium for 21 days. Data are means ± SD (n=9). *P < 0.0001 compared with wild-type MSCs (WT). **c** Changes in osteogenic (ALPL, SPP1) and chondrogenic (ACAN, COL2A1) genes in MSCs with or without the CXCR7 antagonist, CCX771, or agonist, TC14012, incubated with osteogenic or chondrogenic induction medium for 21 days, respectively. Data are means ± SD (n=9). *P < 0.0001 compared with the untreated group (control).

Supplemental figure 2

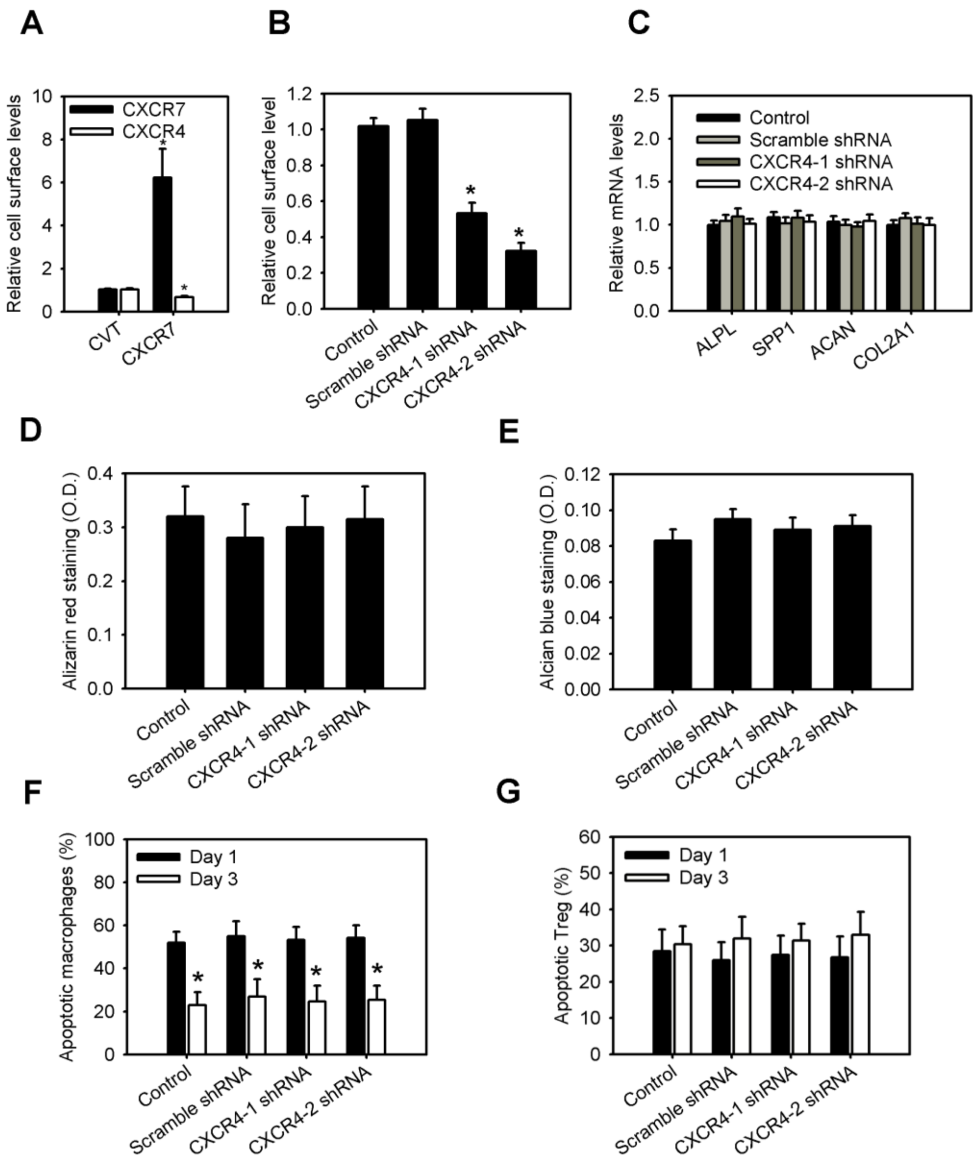

Supplemental figure 2. a Cell-surface expression of CXCR7 and CXCR4 in MSCs lentivirally transduced with control vector (CVT) or CXCR7-expressing vector (CXCR7) for 72 h. Data are means ± SD (n=9). $*P < 0.001$ compared with CVT-expressing MSCs. **b** Verification of CXCR4 knockdown in MSCs lentivirally transduced with the CXCR4 shRNA for 72 h. Data are means ± SD (n=9). $*P <$ 0.0001 compared with wild-type MSCs (control). **c** Changes in osteogenic (ALPL, SPP1) and chondrogenic (ACAN, COL2A1) genes in MSCs with or without CXCR4 knockdown incubated with osteogenic or chondrogenic induction medium for 21 days. Data are means ± SD (n=9). (**d** and **e**) The quantitation of Alizarin red and Alcian blue staining in MSCs with or without CXCR4 knockdown incubated with osteogenic or chondrogenic induction medium for 21 days. Data are means ± SD co-

cultured with MSCs with or without CXCR4 knockdown for 1 or 3 days. Data are means ± SD (n=9). *P < 0.001 compared with the groups on Day 1. (G) Differentiation of regulatory T (Treg, FoxP3^{+} cells)-like cells in Jurkat T cells co-cultured with MSCs with or without CXCR4 knockdown for 1 or 3 days. Data are means ± SD (n=9).

Supplemental figure 3

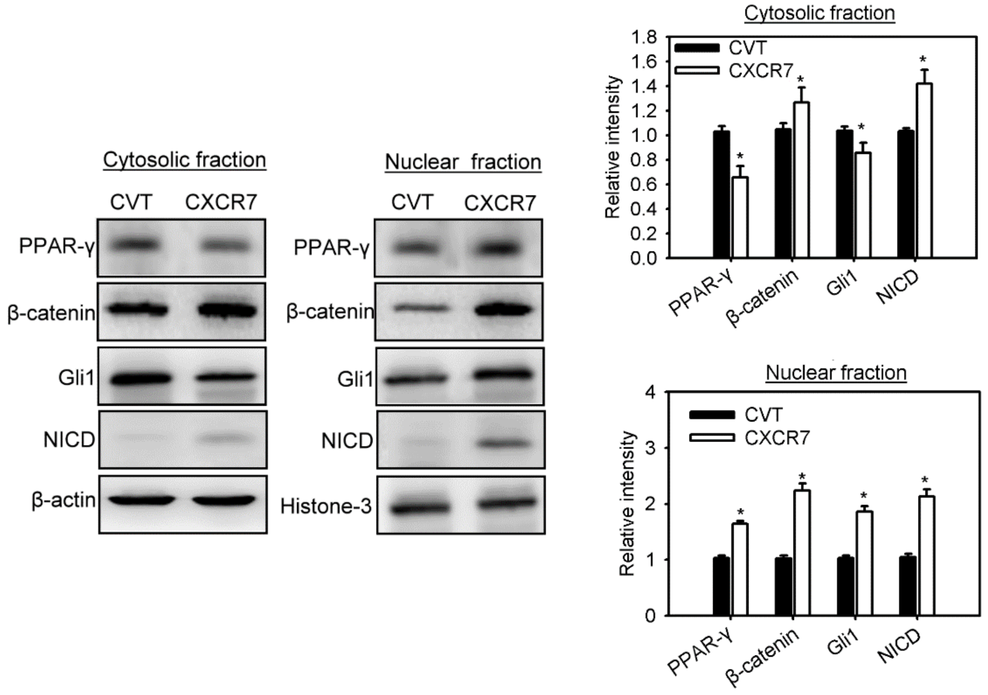

Supplemental figure 3 The localization of PPAR-γ, β-catenin, Gli1 or NICD evaluated by cell fractionation. PPAR-γ, β-catenin, Gli1 and NICD in MSCs lentivirally transduced with control vector (CVT) or CXCR7-expressing vector (CXCR7) for 72 h were detected in both the cytoplasmic and nuclear fractions via western blot analysis. β-actin and Histone-3 were used as internal controls for protein expression in the cytoplasmic and nuclear fractions, respectively. Data are means ± SD (n=9). *P < 0.01 compared with control MSCs (CVT).

Supplemental figure 4

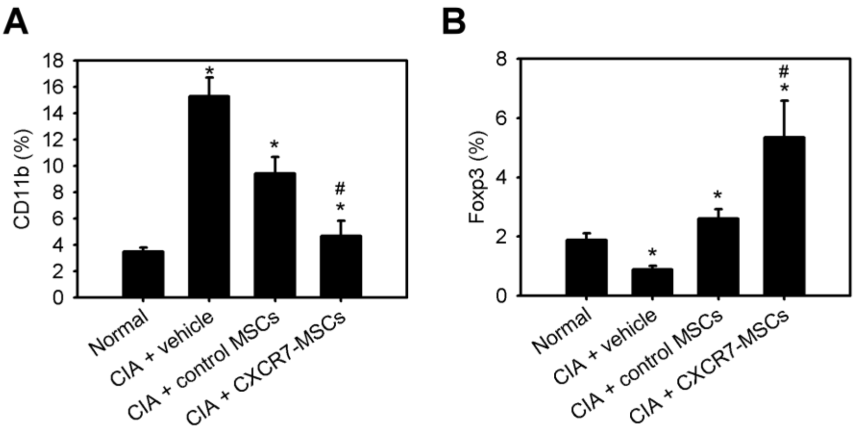

Supplemental figure 4. The frenquency of $CD11b^+$ macrophages (**a**) and $Foxp3^+$ Treg cells (**b**) in synovial tissues derived from normal rats or rats with collagen-induced arthritis (CIA) receiving vehicle (PBS), control vector-expressing MSC (control MSCs) or CXCR7-expressing MSC (CXCR7-MSCs) treatment on day 3. The single cell suspension of synovial tissues were made by enzymatic isolation method and the frenquency of $CD11b^+$ macrophages and $Foxp3^+$ Treg cells were detected by flow cytometry. Data are means ± SD (n=6). *$P < 0.01$ compared with the normal group without CIA. $^{\#}$ $P < 0.01$ compared with the control MSCs.

Supplemental figure 5

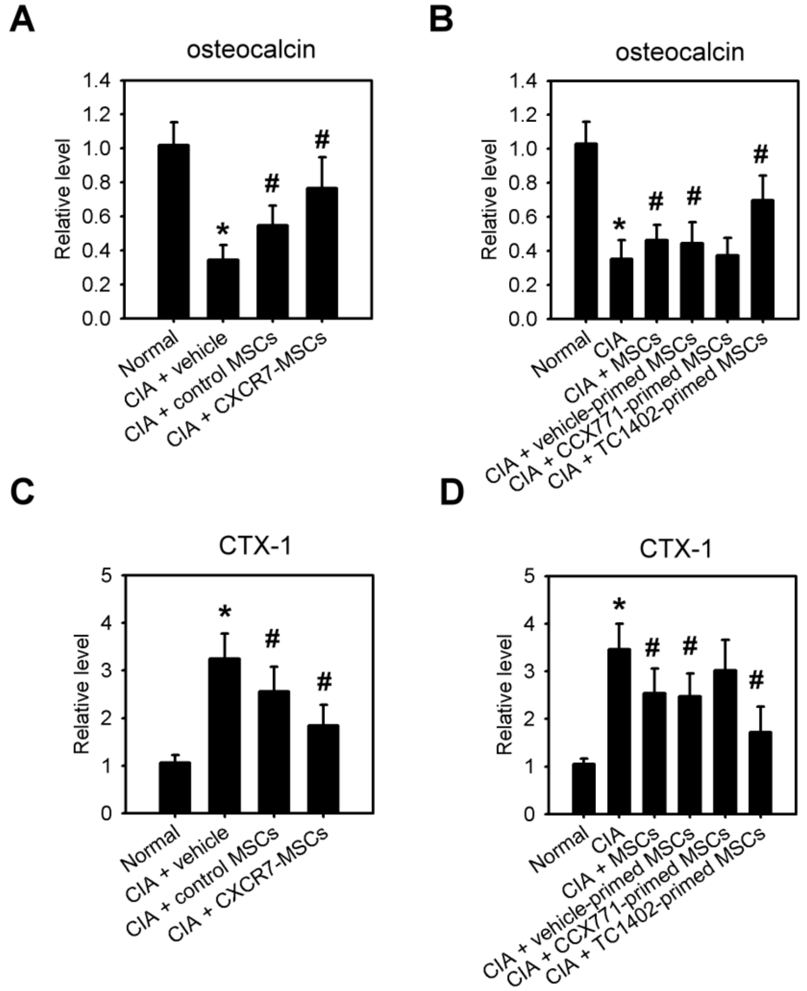

Supplemental figure 5. Levels of osteocaicin (**a** and **b**) and type I collagen cross-linked C-telopeptide (CTX-I, **c** and **d**) in synovial tissues derived from normal rats or rats with collagen-induced arthritis (CIA) receiving vehicle (PBS), control vector-expressing MSC (control MSCs), CXCR7-expressing MSC (CXCR7-MSCs), native MSC (MSCs), vehicle-primed MSC, CCX771-primed MSC or TC14012-primed MSC treatment on Day 28. Data are means ± SD (n=6). *$P < 0.01$ compared with the normal group without CIA. # $P < 0.01$ compared with the CIA group without any treatment.
